# Topological design yields MR-TADF emitters with sub-10 nm emission bandwidths

**DOI:** 10.1093/nsr/nwag468

**Published:** 2026-08-05

**Authors:** Changfeng Si, Eli Zysman-Colman, Zachary M Hudson

**Affiliations:** Department of Chemistry, The University of British Columbia, Canada; Organic Semiconductor Centre, EaStCHEM School of Chemistry, University of St Andrews, UK; Organic Semiconductor Centre, EaStCHEM School of Chemistry, University of St Andrews, UK; Department of Chemistry, The University of British Columbia, Canada

Organic light-emitting diodes (OLEDs) that emit with ultra-high color purity and high external quantum efficiency (EQE) are vital for next-generation ultra-high-definition displays [1,2]. Multi-resonant thermally activated delayed fluorescence (MR-TADF) emitters have emerged as arguably the leading class of materials for narrowband emission, as they typically exhibit photoluminescence spectra with full width at half maximum (FWHM) below 30 nm. This is due to the suppression of vibrational modes arising of their emissive short-range charge transfer excited states. However, further narrowing the emission bandwidth remains challenging because suppressing vibronic coupling often comes at the expense of the orbital delocalization needed to sustain efficient radiative decay [3].

A recent study by Zhang and co-workers introduced a B–N–B bridging motif to construct twisted quadruple borylated MR-TADF emitters [4]. The resulting compound, **2CzBN-BNB**, emits at 501 nm and has an ultra-narrow emission bandwidth in toluene (FWHM = 9 nm), representing the first reported B,N-doped MR-TADF emitter with sub-10-nm FWHM. Vacuum-deposited OLEDs using **2CzBN-BNB** as emitter showed electroluminescence at λ_EL_ = 505 nm with an FWHM of 12 nm and an EQE_max_ of 31.5%. In a related study, Hatakeyama and co-workers recently pushed MR-TADF emitter design to the limit in terms of spectral profile by introducing a modular repetitive architecture that suppresses structural relaxation and vibronic coupling [5]. Their compound, ***m*-CzB10-Mes**, exhibits an unprecedentedly narrow FWHM of 6.9 nm in toluene. The corresponding solution-processed OLEDs using **4tCzBN-PhCN** as a TADF sensitizer showed a moderate EQE_max_ of 18.0% and yielded an ultra-narrow electroluminescence bandwidth of ∼11 nm.

Despite these impressive advances, existing molecular design strategies remain difficult to generalize because each emitter relies on a distinct molecular architecture. Consequently, predicting what structural modifications will best suppress structural relaxation and hence lead to narrowband emission prior to synthesis remains an outstanding challenge. Writing in *National Science Review*, Yang and co-workers address this challenge by introducing molecular topology as a predictive design principle [6]. Rather than optimizing individual molecular structures, the authors systematically enumerated 635 DABNA-derived fusion topologies and identified an alternating C3–A fusion pattern that minimizes excited-state structural relaxation. More importantly, they proposed a simple ground-state descriptor (S2) based on excitation-induced bond-order redistribution, allowing emission bandwidth to be estimated without explicit excited-state calculations. This establishes a set of physically interpretable descriptors linking molecular topology directly to spectral bandwidth (Fig. 1).

**Figure 1.**
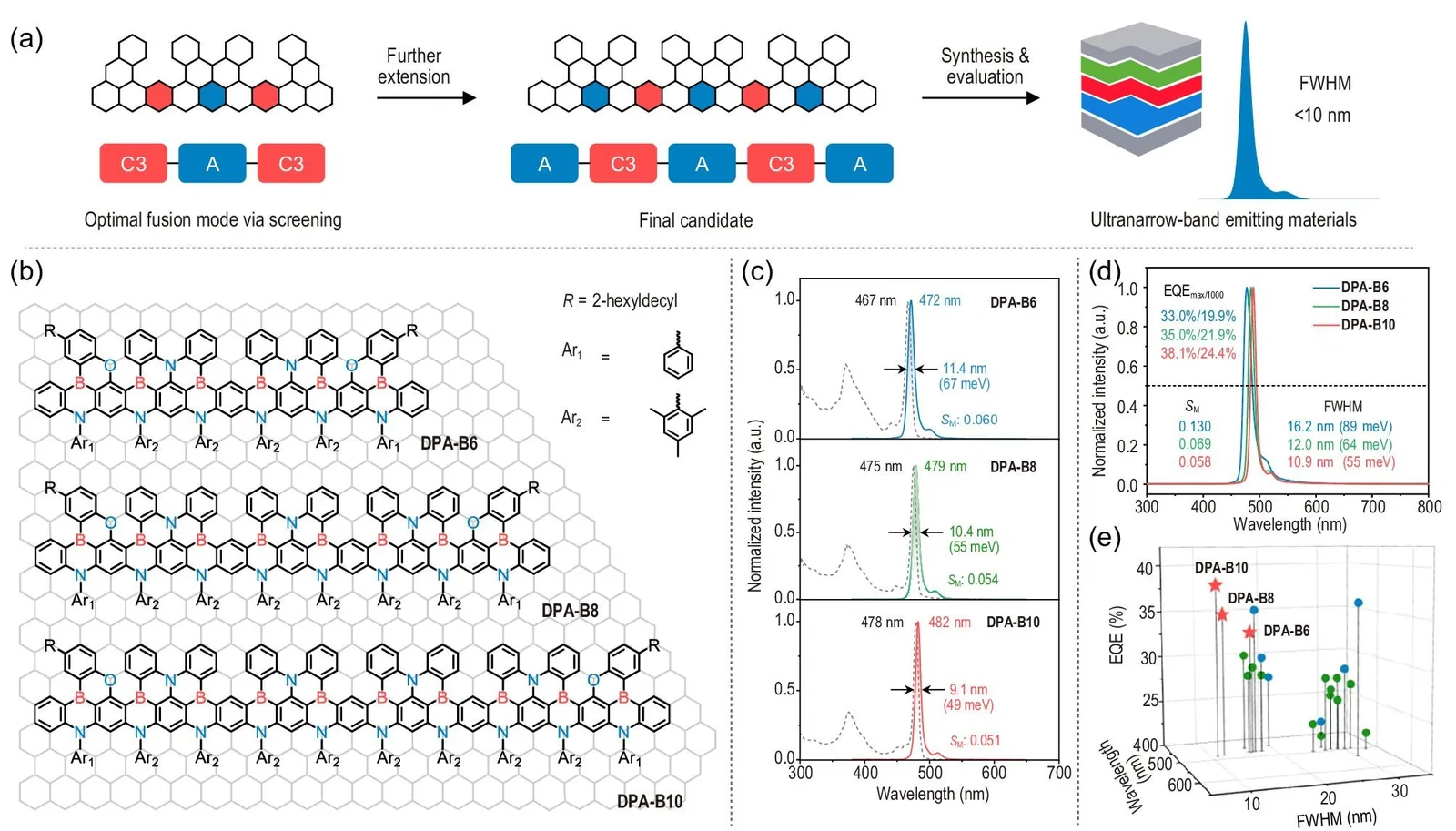
(a) Topology-defined frameworks for ultranarrow-band organic emitters. (b) Chemical structures of DPA-B6, DPA-B8 and DPA-B10. (c) Ultraviolet-visible and fluorescence spectra of the DPA-B*n* series in toluene. (d) Electroluminescent spectra of OLEDs based on DPA-B*n*. (e) Representative solution-processed OLEDs based on MR-TADF emitters. Reproduced from Figs 1–4 of Ref. [6] with permission.

Guided by this framework, the authors systematically extended their design based on their previously reported deep-blue emitter **DPA-B4** [7] into the DPA-B*n* series of compounds (*n* = 6, 8, 10), where *n* denotes the number of boron atoms and the structure incorporates *n*−2 DABNA units. The family exhibits sky-blue fluorescence with emission maxima that gradually red-shift from 472 to 482 nm as the π-system enlarges. Importantly, the emission bands are ultra-narrow and progressively contract across the series, with FWHM values of 11.4 nm (**DPA-B6**), 10.4 nm (**DPA-B8**), and 9.1 nm (**DPA-B10**). In oxygen-free toluene, **DPA-B10** exhibits a near-unity Φ_PL_, a remarkably short delayed lifetime *τ*_d_ of 136.4 ns and a fast *k*_RISC_ of 1.63 × 10^7^ s^−1^, the fastest reported among heavy-element-free TADF emitters. The corresponding solution-processed OLEDs showed an outstanding EQE_max_ of 38.1%, while preserving ultra-narrow electroluminescence with FWHM as small as 10.9 nm. Owing to the fast *k*_RISC_ and large figure of merit values (FOM, a recently proposed metric that combines the radiative decay rate and singlet-triplet equilibrium that is strongly correlated with device efficiency roll-off) [8], efficiency roll-off is partially mitigated, with the DPA-B10 device maintaining an EQE of 34.1% at 100 cd m^−2^ and 24.4% at 1000 cd m^−2^.

This work demonstrates a general design rule—namely, that molecular topologies based on alternating C3–A fusion can minimize excited-state structural relaxation, while the ground-state descriptor S2 provides a practical predictor of emission bandwidth without requiring excited-state calculations. By transforming molecular topology from a structural feature into a predictive design rule, the study provides a rational pathway toward the next-generation printable OLED technologies.
